# The Ventrolateral Periaqueductal Gray Contributes to Depressive-Like Behaviors in Recovery of Inflammatory Bowel Disease Rat Model

**DOI:** 10.3389/fnins.2020.00254

**Published:** 2020-03-24

**Authors:** Chih-Yuan Ko, Ya-Bi Yang, Dylan Chou, Jian-Hua Xu

**Affiliations:** ^1^Department of Respiratory and Critical Care Medicine, The Second Affiliated Hospital, Fujian Medical University, Quanzhou, China; ^2^Department of Clinical Nutrition, The Second Affiliated Hospital, Fujian Medical University, Quanzhou, China; ^3^Respiratory Medicine Center of Fujian Province, Quanzhou, China; ^4^The Sleep Medicine Key Laboratory of Fujian Medical Universities, Fujian Province University, Quanzhou, China; ^5^Physical Examination Center, The Second Affiliated Hospital, Fujian Medical University, Quanzhou, China; ^6^Department of Physiology, Zunyi Medical University, Zhuhai, China; ^7^Department of Tumor Surgery, The Second Affiliated Hospital, Fujian Medical University, Quanzhou, China

**Keywords:** inflammatory bowel diseases, depression, visceral pain, periaqueductal gray, electrophysiology, (2*R*, 6*R*)-hydroxynorketamine

## Abstract

**Background:**

Patients with inflammatory bowel disease (IBD) experience depression, even in the remission phase of IBD symptoms. Although mapping depression-associated brain regions through the gut-brain axis can contribute to understanding the process, the mechanisms remain unclear. Our previous results support the idea that glutamatergic transmission in the ventrolateral periaqueductal gray (vlPAG) mediates stress-induced depression-like behaviors. Thus, we hypothesize that the vlPAG plays a role in regulating depression during remission of IBD.

**Methods:**

We used dextran sulfate sodium (DSS)-induced visceral pain model to evoke depression-like behaviors, assessed by tail suspension test (TST) and sucrose preference test (SPT), and electrophysiological recordings from vlPAG.

**Results:**

Symptoms of animals modeling IBD were relieved by replacing DSS solution with normal drinking water, but their depression-like behaviors sustained. Moreover, the impairment of glutamatergic neurotransmission in vlPAG was sustained as well. Pharmacologically, microinfusion of the glutamate receptor 1 (GluR1) antagonist NASPM into vlPAG mimicked the depression-like behaviors. Furthermore, intra-vlPAG application of AMPA and AMPA receptor-mediated antidepressant (2*R*,6*R*)-hydroxynorketamine [(2*R*,6*R*)-HNK] reversed the DSS-induced depression-like behaviors in the remission phase of visceral abnormalities.

**Conclusion:**

Our results suggest that vlPAG glutamatergic transmission mediates depression-like behaviors during remission of DSS-induced visceral pain, suggesting that vlPAG mapping to the gut-brain axis contributes to depression during remission of IBD.

## Highlights

- DSS evokes short-term visceral abnormalities.
- DSS induces long-term depressive-like behaviors and impairs synaptic transmission in the vlPAG.
- Glutamatergic transmission in the vlPAG contributes to depressive-like behaviors in the remission of IBD-like symptoms.

## Introduction

Inflammatory bowel disease is among the most common gastrointestinal disorder in human, affecting approximately 249–319 per 100,000 of the population in North America (Molodecky et al., 2012). The symptoms of IBD are long-lasting and often combined with other psychological mood disorders, including depression and anxiety (Regueiro et al., 2017; Bruce-Keller et al., 2018; Gracie et al., 2018). Moreover, patients in the remission phase of IBD have high prevalence of anxiety and depression (Simren et al., 2002; Abautret-Daly et al., 2018; Gracie et al., 2018). Establishing effective pharmacological therapies to maintain remission of IBD-related symptoms is a great challenge in the treatment of this disease.

Interestingly, bidirectional regulation of the gut-brain axis is thought to play the role of a relationship in the pathophysiology of gastrointestinal diseases, involving IBD and its related disorders, such as depression and anxiety (Mayer and Tillisch, 2011; Gracie et al., 2018).

The midbrain periaqueductal gray (PAG) is thought to serve as a pain control center (Mayer and Tillisch, 2011) involved in antinociception (Lau and Vaughan, 2014). Moreover, PAG is regulated by peripheral pain, including neuropathic pain (Ho et al., 2013, 2015) and visceral stimuli (Luczynski et al., 2017). The vlPAG, a subarea of the PAG, has been shown to be involved in defensive behaviors, including freezing, flight, and analgesia (Ho et al., 2011, 2013, 2015; Tovote et al., 2016), as well as in stress-induced depressive-like behaviors (Johnson et al., 2016; Chou et al., 2018; Ho et al., 2018). We have demonstrated that diminished GluR1-dependent synaptic transmission in the vlPAG contributes to chronic stress-induced depressive-like behaviors (Ho et al., 2018). In a more recent study, we showed that chronic stress-induced impairment of glutamatergic transmission in the vlPAG was rescued by the antidepressant (2*R*,6*R*)-HNK (Chou et al., 2018).

However, the mechanisms by which IBD map to brain circuits and function through the gut-brain axis, leading to psychological abnormalities, are still largely unknown (Van Oudenhove et al., 2016; Berthoud et al., 2017; Regueiro et al., 2017; Bruce-Keller et al., 2018). Those studies mentioned above raise the possibility that vlPAG glutamatergic neurotransmission plays a role in mediating depressive-like behaviors during remission of IBD. In the present study, we investigated this possibility using a DSS animal model, which is a widely used and reliable model of IBD-like symptoms (Schoellhammer et al., 2017).

## Materials and Methods

### Reagents

Dextran sulfate sodium (#160110; MP Biomedicals), (-) bicuculline methiodide (#2503; Tocris Bioscience), tetrodotoxin (TTX, #1069), (*RS*)-AMPA (#0169; Tocris Bioscience), (2*R*,6*R*)-HNK hydrochloride (#6094; Tocris Bioscience), and 1-naphthyl acetyl spermine trihydrochloride (NASPM; #2766, Tocris Bioscience) dissolved in water or dimethyl sulfoxide (DMSO) were used in the study. The final concentrations of DMSO were controlled to be less than 0.1% in water.

### Animals

Sprague–Dawley adult (6–8 weeks of age) male rats were purchased from Shanghai SLAC Laboratory Animal Co., Ltd. (Shanghai, China). Animals were housed in a temperature- (25 ± 1°C) and humidity-controlled room on a 12-h light/dark cycle (lights on from 06:00 to 18:00) with access to food and water *ad libitum*. The animals were acclimated to the animal research facility for at least 1 week before the start of experiments. All procedures were performed during the light cycle, between 10:00 and 15:00. All experiments were performed in accordance with the guidelines of the Institutional Animal Care and Use Committee of The Second Affiliated Hospital of Fujian Medical University with the certificate number 2019-129. All efforts were made to minimize the number of animals used and the suffering of the animals.

### Dextran Sulfate Sodium Administration

A 5% DSS solution was added to the drinking water for 7 days before replacing with normal drinking water for at least 14 days so the rats could recover, mimicking remission of IBD. During the experiments, a disease activity index score was used to evaluate the progression of colitis, including assessments of stool consistency, stool bleeding, and initial weight loss, as described previously (Chapman et al., 1985). Scores were defined as follows: stool consistency 0 (normal), 2 (soft), or 4 (watery stool); and stool bleeding 0 (no blood), 2 (visual pellet bleeding), or 4 (gross bleeding, blood around anus). Weight loss was calculated and normalized to the initial weight of each individual (day 0). The descending colons were dissected after DSS treatment (day 7), and after replacement of the DSS with regular drinking water (day 21). The colon lengths were measured and compared with those of the control group rats that received only normal drinking water throughout the experimental period.

### Abdominal Withdrawal Reflex

Abdominal withdrawal reflex was used as an index of visceral hypersensitivity, as described previously (Vera-Portocarrero et al., 2003). Rats were first acclimated to the apparatus for 4 h daily for 3 days, and their abdominal hair was shaved. Immediately before the tests, rats were acclimated for at least for 1 h. The sensitivity of the animals was measured before DSS treatment (day 0), after DSS treatment (day 7), and after replacing the DSS solution with regular drinking water (days 14 and 21). Stimuli from von Frey filaments (bending forces from 0.07 to 6 g, with 6 g used as a ceiling) were applied to different points on the abdomen. Withdrawal responses were defined as (1) abdominal withdrawal from the von Frey filament, (2) consequent licking of the abdominal area, or (3) withdrawal of whole body. Reliable and consistent stimuli-responses (subthreshold stimuli-no response and suprathreshold stimuli-withdrawal) that occurred three times were used, and the intensity of suprathreshold stimuli was calculated. The time between stimuli was at least 5 min.

### Colorectal Distension-Induced Visceral Motor Response

Colorectal distension (CRD)-induced visceral motor response (VMR) was used as an index of visceral hypersensitivity, as previously described (Hong et al., 2011; Peng et al., 2014). A PE-100 catheter with a 4-cm deflated flexible latex balloon lubricated with medical-grade lubricant at the tip was inserted intra-anally into the descending colon. The end of the balloon was 1 cm proximal to the anus. Water was infused into the balloon by injecting tube to maintain the intra-colonic pressure (ICP) at 0, 20, 40, or 60 mmHg. ICP was continuously recorded via the catheter connected to a pressure transducer (P23 ID; Gould-Statham, Quincy, IL, United States) on a computer system (MP30; Biopac, Santa Barbara, CA, United States) through a preamplifier (7P1; Grass, Cleveland, OH, United States). The data were recorded and measured using a program built in the recording software Student Lab BSL PRO (version 3.7; Biopac).

For electromyogram recordings, activity was detected by Teflon-coated stainless steel wire electrodes stitched into the external oblique musculature immediately superior to the inguinal ligament. The electromyogram signals were continuously recorded on a computer system (MP30; Biopac) through a preamplifier (P511AC; Grass) using a bandpass filter with a frequency range of 30–3000 Hz. The electromyogram activity was quantified offline by integrating the area under the rectified electromyogram signal using a program built in the recording software Student Lab BSL PRO (ver. 3.7; Biopac).

### Tail Suspension Tests (TSTs)

Tail suspension tests were used to assess despair, a symptom of depression, as previously described (Chou et al., 2014). During the tests, rats were individually suspended by taping the tail to a vertical surface 1 cm from its tip. The total time a rat spent immobile, not expressing escape behavior, was calculated as the index of despair of depression. Rats were considered immobile only when they were passively suspended and remained completely motionless. The immobility time was measured by a blinded observer over the 5-min test.

### Sucrose Preference Tests (SPTs)

Sucrose preference tests were used to assess anhedonia, a symptom of depression, as previously described (Chou et al., 2014, 2018). Rats were first exposed to water with 1% sucrose for 48 h, followed by a 4-h period of water deprivation. During the ensuing test period, each rat was exposed to identical bottles filled with 1% sucrose or water for 1 h. Sucrose solution and water consumption were recorded by measuring the changes in bottle volumes during the test period. Sucrose preference was defined as the ratio of the volume of sucrose solution versus total volume (sucrose solution plus water) consumed during the 1-h test period. The volumes of consumption and ratios were recorded and calculated by a blind observer.

### Whole-Cell Patch-Clamp Recordings

Electrophysiological recordings in the vlPAG slices were conducted as described previously (Ho et al., 2013, 2018). Briefly, 300-μm-thick coronal midbrain PAG slices were dissected from rats. Brain slices were then equilibrated in artificial cerebral spinal fluid (aCSF) at room temperature for at least 1 h before electrophysiological recordings were taken. The aCSF contained 117 mM NaCl, 4.5 mM KCl, 2.5 mM CaCl_2_, 1.2 mM MgCl_2_, 1.2 mM NaH_2_PO_4_, 25 mM NaHCO_3_, and 11.4 mM dextrose, and was oxygenated with 95%/5% O_2_/CO_2_ (pH 7.4). Visualized whole-cell patch-clamp recordings were conducted under a stage-fixed upright IR-DIC microscope (BX51WI; Olympus, Tokyo, Japan) equipped with a 40 × water-immersion objective.

Synaptic currents were recorded with 4–6-MΩ microelectrodes filled with a Cs^+^-based internal solution containing 110 mM Cs^+^ gluconate, 5 mM TEA, 5 mM QX314, 0.5 mM CaCl_2_, 5 mM BAPTA, 10 mM HEPES, 5 mM MgATP, 0.33 mM GTP-Tris (pH 7.3), and 280 mOsm/L (liquid junction potential = 14.6 mV). Recording microelectrodes were filled with a Cs^+^-based internal solution, except in experiments measuring neuronal excitability, in which a K^+^-based internal solution was used. The K^+^-based internal solution contained 125 mM K^+^ gluconate, 5 mM KCl, 0.5 mM CaCl_2_, 5 mM BAPTA, 10 mM HEPES, MgATP 5, 0.33 mM GTP-Tris (pH 7.3), and 280 mOsm/L (liquid junction potential = 11.4 mV). Excitatory postsynaptic currents (EPSCs) and miniature EPSCs (mEPSCs) were recorded at −70 mV in the presence of (-)-bicuculline methiodide (10 μM), a GABAA receptor antagonist. Spontaneous mEPSCs were recorded in the presence of TTX (1 μM), a sodium channel blocker. Neurons with larger membrane capacitance (40–100 pF) and lower membrane resistance (250–750 MΩ) were recorded in the present study because neurons with these two passive membrane properties are more likely to be glutamatergic projection neurons in the vlPAG (Park et al., 2010; Ho et al., 2011).

Excitatory postsynaptic currents were evoked at 0.1 Hz by 150-μs-width pulses from a Grass stimulator (Grass Telefactor S88; W. Warwick, RI, United States) through a bipolar concentric electrode (Frederick Haer & Co., United States) placed 50–150 μm away from the recording electrode. Paired-pulse ratios (PPRs) of EPSCs were recorded with pulses of 50 ms given every 20 s. PPR was defined as the ratio of the averaged amplitude of the second EPSC/IPSC (EPSC2/IPSC2) to that of the first EPSC/IPSC (EPSC1/IPSC1).

All electrophysiological signals were acquired and analyzed using an Axon setup (Molecular Device/Axon Instruments, Foster City, CA, United States). Signals were sampled at 5–10 kHz by pClamp 9.2 with an Axopatch 200B amplifier and Digidata 1322A AD-converter, and analyzed by Clampfit 9.2. The access resistance was monitored continuously throughout the recordings and neurons were discarded if the access resistance changed by > 15%.

### Brain Cannula Implantation and Microinjection

Brain cannula implantations and microinjections were performed with standard procedures, as described previously (Chou et al., 2018). Rats were first anesthetized with isoflurane (5% for induction, 2% for maintenance) and placed in a stereotaxic frame (Stoelting, Wood Dale, IL, United States). A 24-gauge, 12-mm stainless steel guide cannula was implanted stereotaxically into the vlPAG (AP: 7.8 mm; L: 0.5 mm; DV: 5.8 mm). Rats were allowed to recover for at least 7 days before the start of the experiments. For microinjection, drugs were microinfused into the vlPAG through a 30-gauge injection cannula via a 1 μL-Hamilton syringe connected to a microinfusion pump (KDS311; KD Scientific Inc., Holliston, MA, United States). A 0.2-μL drug solution was slowly infused into the vlPAG over 2 min before waiting for 3 min to prevent backflow of the drug. At the end of the experiments, the rats used in the behavioral tests were sacrificed by perfusion and the injection sites were evaluated for each animal. Only those rats that had accurate injections were chosen for analysis.

### Statistical Analyses

The data in this study were analyzed using the Prism 5 software package (GraphPad software) and are expressed as mean ± standard error of mean (SEM). Data for time-course experiments were analyzed by repeated measure two-way analysis of variance (ANOVA). Data with two factors were analyzed by two-way ANOVA and those with one factor were analyzed by one-way ANOVA. Bonferroni’s *post hoc* analyses were used to compare the means of the groups. In other cases, unpaired Student’s *t*-tests were used to compare the means of groups. Significance was set at *p* < 0.05.

## Results

### Establishing Remission in an IBD Animal Model

**FIGURE 1:**
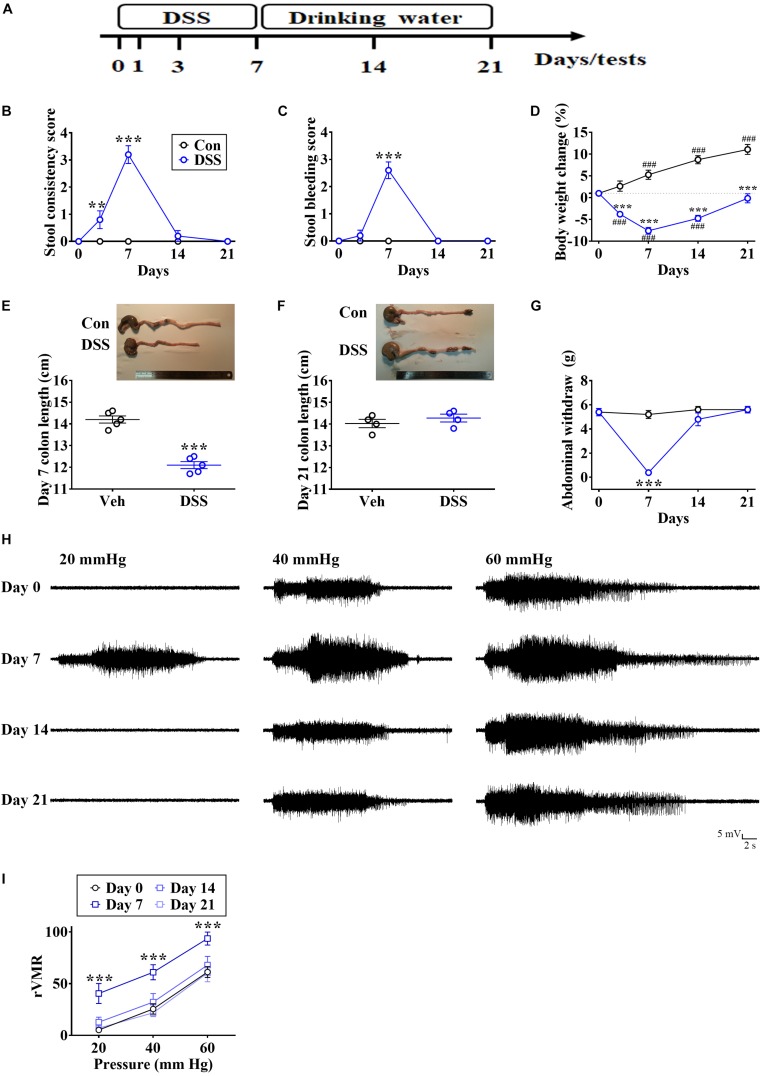
DSS-evoked short-term visceral abnormalities. **(A)** Experimental design for treatment with DSS solution, which was later replaced by normal drinking water. **(B,C)** Summary of experiments showing time profiles of stool consistency and bleeding score in DSS-treated and control (Con) rats. **(D)** Summary of experiments showing changes in body weight over time in DSS-treated and Con rats. **(E,F)** Image and vertical scatterplot demonstrating colon lengths on days 7 and 21 in DSS-treated and Con rats. **(G–I)** Summary of experiments and representative recording traces illustrating DSS-induced visceral hypersensitivity in von Frey filaments-induced abdominal withdrawal and CRD-induced VMR. Statistics were analyzed by repeated measure two-way ANOVA followed by *post hoc* Bonferroni’s test and unpaired Student’s *t*-test. Data are represented as mean ± SEM. **p < 0.01, ***p < 0.001, comparing with Con. ^###^*p* < 0.001, compared with day 0.

Rats first received DSS dissolved in drinking water, which was later replaced by normal drinking water to promote recovery (Figure 1A). IBD-like symptoms included increased stool consistency (Figure 1B, Time: *F*_4_,_36_ = 42.43, *p* < 0.0001; Treatment: *F*_1_,_9_ = 44.6, *p* < 0.0001; Interaction: *F*_4_,_36_ = 42.43, *p* < 0.0001; *n* = 10 in each group, repeated measure two-way ANOVA), appearance of blood in the stool (Figure 1C, Time: *F*_4_,_36_ = 55.53, *p* < 0.0001; Treatment: *F*_1_,_9_ = 40.09, *p* < 0.001; Interaction: *F*_4_,_36_ = 55.53, *p* < 0.0001; *n* = 10 in each group, repeated measure two-way ANOVA), decreased body weight (Figure 1D, Time: *F*_4_,_36_ = 41.53, *p* < 0.0001; Treatment: *F*_1_,_9_ = 85.84, *p* < 0.0001; Interaction: *F*_4_,_36_ = 66.52; *p* < 0.0001, *n* = 10 in each group, repeated measure two-way ANOVA), and decreased colon length (Figure 1E, *t* = 9.209, *p* < 0.0001, unpaired Student’s *t*-test) on day 7, compared with the control (Con). There were no differences after the recovery on days 14 and 21 (Figures 1B,C,F).

Visceral hypersensitivity is recognized as a crucial symptom of IBD in patients and animals (Delvaux, 2002; Park et al., 2010; Luczynski et al., 2017). Therefore, we examined the visceral sensitivity by von Frey filament-induced abdominal withdrawal and by CRD-induced VMR. We found that DSS treatment dramatically decreased the abdominal withdrawal threshold (Figure 1G, Time: *F*_3_,_27_ = 26.82, *p* < 0.0001; Treatment: *F*_1_,_9_ = 35.81, *p* = 0.0002; Interaction: *F*_3_,_27_ = 30.95, *p* < 0.0001; *n* = 10 in each group, repeated measure two-way ANOVA) and enhanced CRD-induced VMR (Figures 1H,I, Time: *F*_3_,_54_ = 26.27, *p* < 0.0001; Stimuli intensity: *F*_2_,_18_ = 43.95, *p* < 0.0001; Interaction: *F*_6_,_54_ = 0.07832, *p* = 0.998; *n* = 7 in each group, repeated measure two-way ANOVA) on day 7, and no significant differences were observed after the recovery on days 14 or 21 (Figures 1G–I).

### DSS Treatment Induces Long-Lasting Behavioral Changes

**FIGURE 2:**
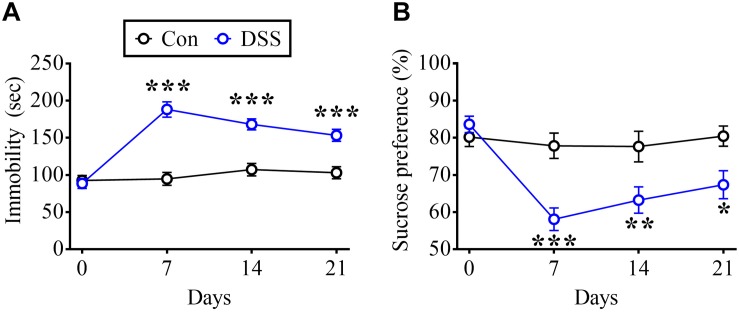
DSS-induced long-term depressive-like behaviors. **(A)** Summary of experiments showing time profile of rats displaying immobilized behavior in TSTs in DSS-treated and Con rats. **(B)** Summary of experiments indicating time profile of preference in SPTs in DSS and Con rats. Statistics were analyzed by repeated measure two-way ANOVA followed by *post hoc* Bonferroni’s test. Data are represented as mean ± SEM. *p < 0.05, **p < 0.01, ***p < 0.001 comparing with Con.

To test the behavioral performances during remission of IBD model animals, DSS-treated rats not only exclaimed by TSTs also measured by SPTs. DSS treatment significantly increased the time in the immobilized state during TSTs on days 7, 14, and 21 (Figure 2A, Time: *F*_3_,_54_ = 21.83, *p* < 0.0001; Treatment: *F*_1_,_18_ = 44.6, *p* < 0.0001; Interaction: *F*_3_,_54_ = 16.57, *p* < 0.0001; *n* = 10 in each group, repeated measure two-way ANOVA). Moreover, DSS treatment dramatically decreased the preference for sucrose in SPTs on day 7, day 14, and day 21 (Figure 2B, Time: *F*_3_,_54_ = 6.711, *p* = 0.0006; Treatment: *F*_1_,_18_ = 28.08, *p* < 0.0001; Interaction: *F*_3_,_54_ = 4.562, *p* = 0.0064; *n* = 10 in each group, repeated measure two-way ANOVA).

### DSS Treatment Causes Long-Lasting Impairment of Glutamatergic Transmission in the vlPAG

**FIGURE 3:**
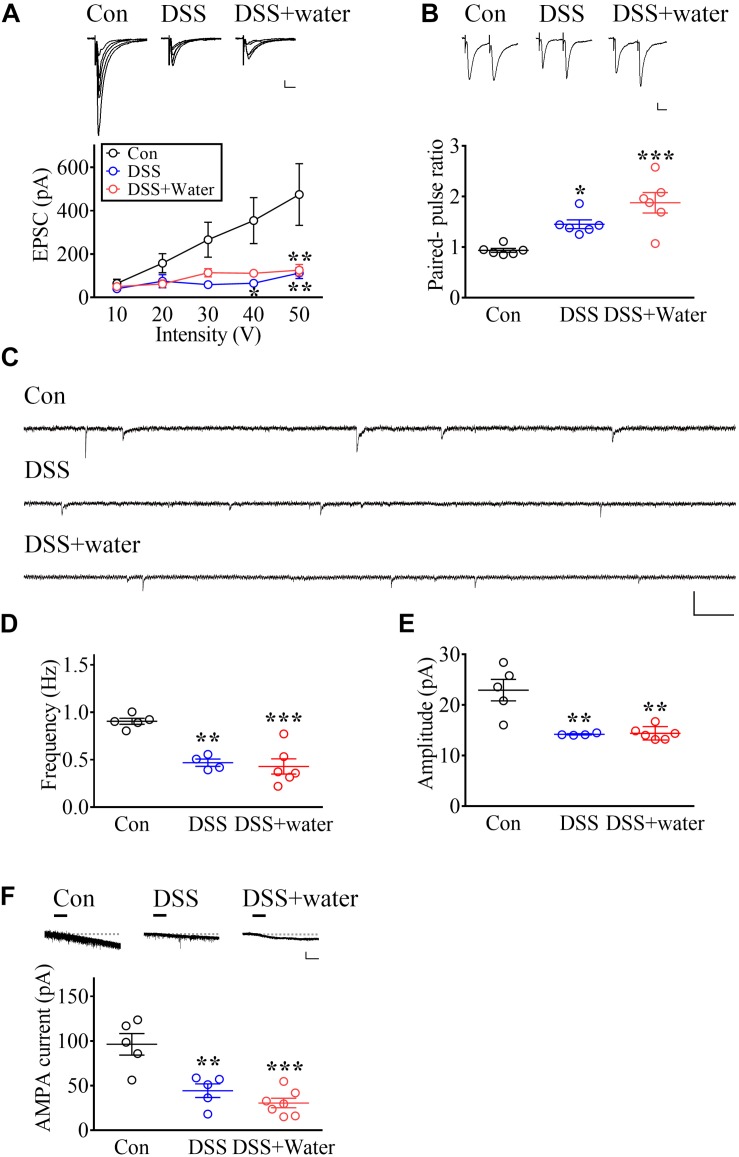
Long-lasting impairment of glutamatergic transmission in vlPAG by DSS treatment. **(A)** Representative traces of electrophysiological recordings and summary of experiments illustrating the input–output curve in vlPAG neurons obtained from Con (Day 0), DSS (Day 7), and later drinking water replacement (DSS + water; day 21). Calibration: 20 pA, 20 ms. **(B)** Representative recording traces and vertical scatterplot depicting the distribution of PPRs in vlPAG neurons evoked by two identical electric stimuli with 50 ms inter-pulse intervals, obtained from the Con, DSS, and DSS + water groups. Calibration: 20 pA, 20 ms. **(C)** Representative traces showing the mEPSCs recorded in the vlPAG obtained from the Con, DSS, and DSS + water groups. Calibration: 30 pA, 300 ms. **(D,E)** Vertical scatterplot depicting the distribution of the frequency and amplitude of mEPSCs in the Con, DSS, and DSS + water groups. **(F)** Representative traces and scatterplot showing AMPA (1 μM)-evoked inward currents in vlPAG neurons. Horizontal bars denote that AMPA was applied. The dashed line indicates the baseline. Calibration: 50 pA, 30 s. Statistics were analyzed by one-way and repeated measure two-way ANOVA followed by *post hoc* Bonferroni’s test. Data are represented as mean ± SEM. *p < 0.05, **p < 0.01, ***p < 0.001, comparing with Con.

Based on our previous studies, diminished vlPAG glutamatergic transmission contributes to chronic stress-induced depressive-like behaviors (Chou et al., 2018; Ho et al., 2018). Thus, we investigated the role of vlPAG synaptic transmission in DSS-induced sustained depression. First, we assessed the impact of DSS treatment on basal synaptic transmission in the vlPAG by measuring current–voltage relationship of EPSCs in the vlPAG neurons evoked by electrical stimulation at various holding potentials. The EPSC amplitude of input–output curves was reduced in both DSS-treated (day 7) and DSS + water (day 21) groups (Figure 3A, Stimuli intensity: *F*_4_,_60_ = 5.795, *p* = 0.0005; Groups: *F*_2_,_15_ = 2.627, *p* = 0.1052; Interaction: *F*_8_,_60_ = 2.902, *p* = 0.0084; Con: *n* = 9; DSS: *n* = 4; DSS + water: *n* = 5, repeated measure two-way ANOVA).

To further investigate whether presynaptic mechanisms contributed to impairments, we examined PPRs. Results showed that both the DSS and DSS + water groups had enhanced PPRs (Figure 3B, *F*_2_,_15_ = 13.38, *p* = 0.0005; *n* = 6 in each group, one-way ANOVA), suggesting that a presynaptic mechanism might contribute to the impairment of synaptic transmission. Moreover, we investigated the mEPSCs in the vlPAG. The frequency (Figures 3C,D, *F*_2_,_12_ = 18.52, *p* = 0.0002; Con: *n* = 5; DSS: *n* = 4; DSS + water: *n* = 6, one-way ANOVA) and the amplitude (Figures 3C,E, *F*_2_,_12_ = 14.95, *p* = 0.0006; Con: *n* = 5; DSS: *n* = 4; DSS + water: *n* = 6, one-way ANOVA) of mEPSCs were decreased in both the DSS and DSS + water groups. These results suggest that both presynaptic and postsynaptic mechanisms contribute to the impairments.

We next examined whether postsynaptic mechanisms contributed to the impairments and found that AMPA-mediated currents in the vlPAG were depressed in both the DSS and DSS + water groups (Figure 3F, *F*_2_,_14_ = 17.62, *p* = 0.0002; Con: *n* = 5; DSS: *n* = 5; DSS + water: *n* = 7, one-way ANOVA).

### vlPAG-Mediated Depressive-Like Behaviors in the Remission of DSS-Induced Visceral Abnormalities

**FIGURE 4:**
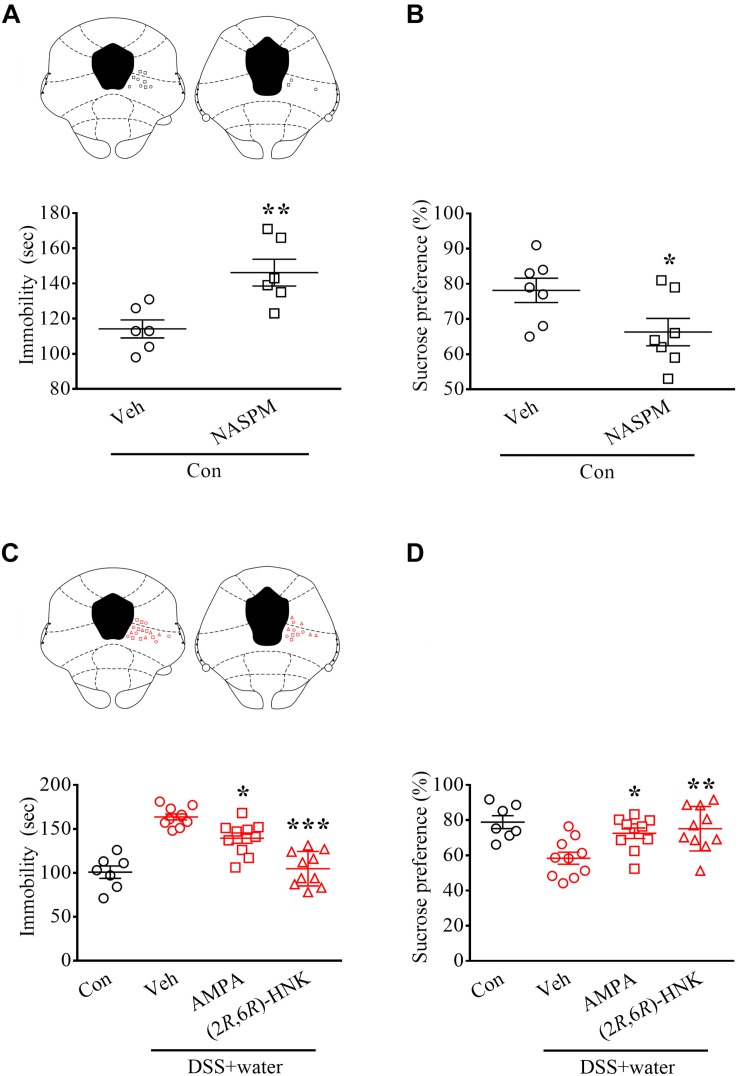
Glutamatergic transmission in vlPAG-mediated depressive-like behaviors during remission of DSS-induced visceral abnormalities. **(A,B)** Vertical scatterplot showing the distribution of the immobility in the TSTs and the preference in the SPTs. Naïve Con rats received either vehicle (Veh) or the selective GluR1 antagonist NASPM (5 μg/0.2 μL) via pre-implanted cannula targeting the vlPAG 1 h before the tests. The histology of the vlPAG representing each injecting site. **(C,D)** Scatterplot showing the performances of the Con and DSS + water (day 21) groups in the TSTs and SPTs. Rats received Veh, AMPA (0.05 μg/0.2 μL), or (2*R*,6*R*)-HNK (1 pg/0.2 μL) via pre-implanted cannula 1 h before the tests. The histology of the vlPAG represented each injecting site. Statistics were analyzed by one-way ANOVA followed by *post hoc* Bonferroni’s test and unpaired Student’s *t*-test. Data are represented as mean ± SEM. *p < 0.05, **p < 0.01, ***p < 0.001, comparing with Veh.

To determine whether decreased AMPA function in the vlPAG contributes to DSS-induced animal depression, we microinjected the selective GluR1 antagonist NASPM into the vlPAG of naïve Con rats. As previously reported (Chou et al., 2018), intra-vlPAG application of NASPM increased immobility in TSTs (Figure 4A, *t* = 3.489, *p* = 0.0058; *n* = 6 in each group, unpaired Student’s *t*-test) and decreased the sucrose preference in SPTs (Figure 4B, *t* = 2.284, *p* = 0.0414; *n* = 7 in each group, unpaired Student’s *t*-test). These results suggest that inhibition of GluR1 AMPA receptor functions in the vlPAG is sufficient to evoke depressive-like behaviors.

Moreover, we directly microinfused AMPA and the AMPA receptor-mediated antidepressant (2*R*,6*R*)-HNK into the vlPAG, which reversed DSS-induced enhancement of immobility in the TSTs [Figure 4C, *F*_2_,_27_ = 31.17, *p* < 0.0001; *n* = 7 in Con and *n* = 10 in Veh, AMPA, and (2*R*,6*R*)-HNK, one-way ANOVA] and blocked DSS-induced inhibition of preference in the SPTs [Figure 4D; *F*_2_,_27_ = 6.67, *p* = 0.0044; *n* = 7 in Con and *n* = 10 in Veh, AMPA, and (2*R*,6*R*)-HNK, one-way ANOVA] in the DSS + water group.

## Discussion

Our previous study reported that reduced glutamatergic transmission in the vlPAG contributes to chronic stress-induced depressive-like behaviors (Chou et al., 2018). The present results provide that DSS impairs vlPAG glutamatergic transmission, even after recovery from visceral abnormalities. The time profiles of the DSS-induced impairments of synaptic transmission in the vlPAG are highly associated with DSS-evoked increased the immobilized times in TSTs and decreased the sucrose preference in SPTs. Moreover, restoring vlPAG glutamatergic transmission by intra-vlPAG application of AMPA and (2*R*,6*R*)-HNK eliminated DSS-induced long-term behavioral changes (the immobilized times reduced in TSTs and the sucrose preference increased in SPTs). The present study suggests that vlPAG glutamatergic transmission mediates depressive-like behaviors in the remission of DSS-induced IBD-like symptoms.

Additionally, anxiety symptoms have also been observed during remission in IBD patients (Abautret-Daly et al., 2018). Similarly, we found that DSS did evoke long-term anxiety-like behaviors in our model animals; however, AMPA micro-infused into the vlPAG did not alter DSS-induced anxiety-like behaviors (Supplementary Material). Evidence indicates that DSS enhances the expression of various genes, including brain-derived neurotrophic factor, in the amygdala, hippocampus, and hypothalamus (Reichmann et al., 2015). Notably, these brain regions are highly associated with anxiety. It is putatively implied that DSS mapping the vlPAG to alter depressive-like behaviors and mapping those brain regions to influence anxiety-like behaviors.

The ketamine metabolite (2*R*,6*R*)-HNK was recently demonstrated to relieve depression in animals with rapid onset and long-lasting action (Zanos et al., 2016; Chou et al., 2018), and is considered a next-generation antidepressant candidate (Zanos et al., 2016). The antidepressant activity of (2*R*,6*R*)-HNK occurs through increasing AMPA receptor-dependent activity, and does not produce any of the side effects of ketamine (Zanos et al., 2016). In the present study, intra-vlPAG application of (2*R*,6*R*)-HNK significantly reversed DSS-induced long-term depressive-like behaviors, strengthening the evidence that vlPAG helps mediate animal depression. Notably, an explanation for TSTs or forced swim tests (FSTs) might not an ideal model of depression, it is thought as an adaptive behavior coping with the stressful condition (Molendijk and de Kloet, 2019).

The PAG receives high brain region inputs, including from the medial prefrontal cortex, anterior cingulate cortex, amygdala, and hypothalamus (Heinricher et al., 2009), and receives pain information from spinal neurons via the spinomesencephalic ascending tract (Basbaum et al., 2009). Results from the present study indicate that DSS-induced visceral pain impairs vlPAG glutamatergic input transmission. Future studies are needed to further clarify which neuronal circuit inputs contribute to DSS-induced impairment of synaptic transmission.

The PAG plays a crucial role in pain regulation through descending projections to the rostral ventral ventromedial medulla (Heinricher et al., 2009). In addition to pain modulation, the current study extends PAG function to mediating depression (Ho et al., 2018), perhaps through the control of dopaminergic neurons in the ventral tegmental area, leading them to ascendingly innervate high brain regions (Moloney et al., 2015; Ntamati et al., 2018). It is likely that the PAG acts as a relay center, exchanging information on visceral pain and depression, in which impairment of PAG glutamatergic transmission in pain mapping leads to depression, and relieving the impairment during pain remission leads to psychological well-being. Thus, as impairment of PAG glutamatergic transmission still occurs during remission of visceral pain, the possibility of additional mechanisms, such as epigenetic regulation, requires further investigation.

Mounting evidence suggests that gut microbiota is altered in IBD, leading to changes in brain function via the gut-brain axis and, interestingly, an enlarged PAG is involved in pain processing (Luczynski et al., 2017; Humbel et al., 2019). Notably, administration of probiotics in patients with gastrointestinal disorders was found to decrease their score of depression and to alter depression-associated brain activity (Pinto-Sanchez et al., 2017). Future work is needed to clarify the role of PAG in microbiota-induced brain mapping and in related diseases.

## Conclusion

The present study indicates that DSS-induced visceral pain maps to the vlPAG by decreasing glutamatergic neurotransmission, which mediates depressive-like behaviors during remission of an IBD model in rodents. Our findings provide new insights into vlPAG glutamatergic transmission underlying the gut-brain axis and its involvement in visceral pain-induced psychological disorders.

## Data Availability Statement

All datasets generated for this study are included in the article/Supplementary Material.

## Ethics Statement

The animal study was reviewed and approved by The Second Affiliated Hospital of Fujian Medical University with the certificate number 2019-129.

## Author Contributions

C-YK and J-HX participated in the design of the study, writing the protocol. Y-BY and DC carried out behavioral analyses and cared for rats. C-YK, Y-BY, and J-HX conducted kinds of literature searches and data analyses and writing drafts of the manuscript.

## Conflict of Interest

The authors declare that the research was conducted in the absence of any commercial or financial relationships that could be construed as a potential conflict of interest.

## Abbreviations

AMPA: α-amino -3-hydroxy-5-methyl-4-isoxazolepropionic acid
DSS: dextran sulfate sodium
IBD: inflammatory bowel disease
GluR1: glutamate receptor 1
(2*R*,6*R*)-HNK: (2*R*,6*R*)-hydroxynorketamine
SPT: sucrose preference test
TST: tail suspension test
vlPAG: ventrolateral periaqueductal gray.
