# Supplementary material for: The Ventrolateral Periaqueductal Gray Contributes to Depressive-Like Behaviors in Recovery of Inflammatory Bowel Disease Rat Model
Source: Front Neurosci. 2020 Mar 24;14:254. doi: 10.3389/fnins.2020.00254 (PMC7105903; doi:10.3389/fnins.2020.00254)
Supplement: Supplementary file 1 [file Table_1.DOCX]

**Supplementary data**


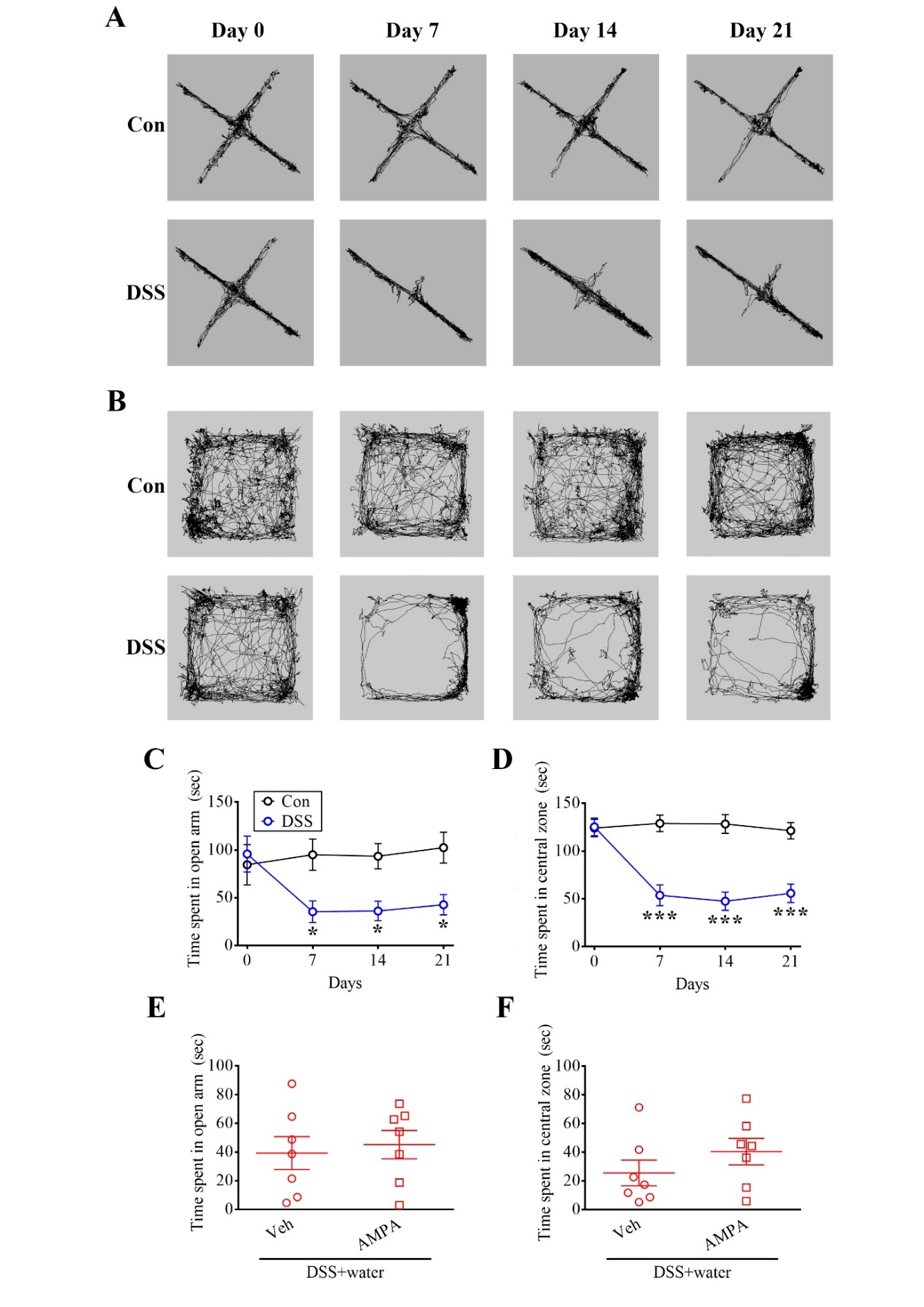


**Supplementary figure 1. Intra-vlPAG application of AMPA did not alter DSS-induced long-term anxiety-like behaviors.** (A) Representative recording traces of elevated plus maze (EPM) obtained from Con (day 0), DSS (day 7), and DSS + water (day 14 and 21) groups, comparing with drinking water-treated alone Con. (B) Representative examples of open field test (OPT) obtained from Con, DSS, and DSS + water groups, comparing with Con. (C) Summary of experiments showing time profile of rats spent their time in open arm of the EPM in DSS-treated and Con groups. [Time: F_3,78_ = 1.32, p = 0.2739, Treatment: F_1,26_ = 12.34, p = 0.0016, Interaction: F_3,78_ = 2.88, p = 0.0412]. (D) Summary of experiments illustrating time profile of the time spent in central zone of the OPT in DSS-treated and Con groups. [Time: F_3,78_ = 7.836, p = 0.0001, Treatment: F_1,26_ = 53.61, p < 0.0001, Interaction: F_3,78_ = 8.979, p < 0.0001]. (E) Vertical scatterplot indicating the distribution of intra-vlPAG application of α-amino-3-hydroxy-5-methyl-4-isoxazolepropionic acid (AMPA) or vehicle (Veh) 1 hour before the EPM test in the DSS + water group (day 21) [t_(12)_ = 0.3908, p = 0.7028]. (F) Vertical scatterplot demonstrating the distribution of intra-vlPAG application of AMPA or Veh 1 hour before the OPT in the DSS + water group [t_(12)_ = 1.164, p = 0.267]. Data are presented as mean ± SEM, n = 14 in each group. Statistics were analyzed by repeated measure two-way ANOVA followed by post hoc Bonferroni’s test and unpaired Student’s *t* test. **P* < 0.05, ****P* < 0.001, comparing with Con.
